# Digestive enzymes and gut morphometric parameters of threespine stickleback (*Gasterosteus aculeatus*): Influence of body size and temperature

**DOI:** 10.1371/journal.pone.0194932

**Published:** 2018-04-03

**Authors:** Younes Mohamed Ismail Hani, Adrien Marchand, Cyril Turies, Elodie Kerambrun, Olivier Palluel, Anne Bado-Nilles, Rémy Beaudouin, Jean-Marc Porcher, Alain Geffard, Odile Dedourge-Geffard

**Affiliations:** 1 Unité mixte de recherche Stress Environnementaux et Biosurveillance des milieux aquatiques (UMR-I 02 SEBIO), Reims, France; 2 Institut National de l’Environnement Industriel et des Risques (INERIS), Unité d’Ecotoxicologie in vitro et in vivo (ECOT), Verneuil-en-Halatte, France; 3 Université de Reims Champagne Ardenne (URCA), Moulin de la Housse, Reims, France; 4 Institut National de l’Environnement Industriel et des Risques (INERIS), Unité Modèles pour l’Ecotoxicologie et la Toxicologie (METO), Verneuil-en-Halatte, France; Institute of Zoology, CHINA

## Abstract

Determining digestive enzyme activity is of potential interest to obtain and understand valuable information about fish digestive physiology, since digestion is an elementary process of fish metabolism. We described for the first time (i) three digestive enzymes: amylase, trypsin and intestinal alkaline phosphatase (IAP), and (ii) three gut morphometric parameters: relative gut length (RGL), relative gut mass (RGM) and Zihler’s index (ZI) in threespine stickleback (*Gasterosteus aculeatus*), and we studied the effect of temperature and body size on these parameters. When mimicking seasonal variation in temperature, body size had no effect on digestive enzyme activity. The highest levels of amylase and trypsin activity were observed at 18°C, while the highest IAP activity was recorded at 20°C. When sticklebacks were exposed to three constant temperatures (16, 18 and 21°C), a temporal effect correlated to fish growth was observed with inverse evolution patterns between amylase activity and the activities of trypsin and IAP. Temperature (in both experiments) had no effect on morphometric parameters. However, a temporal variation was recorded for both RGM (in the second experiment) and ZI (in both experiments), and the later was correlated to fish body mass.

## Introduction

The energy metabolism is the main and most important biological parameter involved in all physiological processes of lower vertebrates. Their energy requirements are derived from the oxidation of organic compounds (carbohydrates, lipids and proteins) produced from food digestion [1]. Digestion is an elementary process in fish metabolism because it determines the availability of nutrients needed for all biological concerns [2]. Digestive enzymes are one of the cornerstones of digestion for the net efficiency of the whole digestive process mainly depends on the type and functional characteristics of these parameters [3]. Degradation of nutrients in the digestive tract of fish largely depends on available enzymes. Hence, determining digestive enzyme activity is of potential interest to obtain and complete valuable information concerning fish digestive physiology. Digestive enzymes are extensively studied in aquaculture. Thus, studies on digestive secretions have helped to define the limits of dietary utilization of proteins and carbohydrates and supported the development of nutritional strategies for fish feeding and diet formulation [4]. In all aquatic species, as in fish, digestive enzyme activity is indicative of their feeding ecology and trophic niche in natural conditions and are well correlated with their diet [5]. In addition, digestive enzymes have also been investigated in invertebrate species (*i*.*e*. *Gammarus sp*.*)* as potential biomarkers in aquatic ecotoxicology [6].

Digestive enzyme activity varies within species. It is influenced by biotic (size, age, origin), and abiotic (temperature, season, food) parameters [7], which can alter enzyme profiles or activity levels. Based on their food preferences, fish are conventionally classified as carnivorous, herbivorous or omnivorous [3]. They can also be classified according to their gut morphometric parameters, *i*.*e*. relative gut mass (RGM), relative gut length (RGL), and Zihler’s Index (ZI, the relation between gut length and ten times the cube root of body mass) [8, 9]. RGL and ZI have been investigated as potential indices of fish dietary strategy based on gut length [8, 10]. RGM (also called digestive somatic index) has been used to determine the feeding states of fish and can give an estimation of the amount of tissue dedicated by fish to their digestive tract [8].

Most fish possess seven main digestive enzymes, classified as proteolytic enzymes (*i*.*e*. trypsin, carboxypeptidase a, carboxypeptidase b), carbohydrate enzymes (*i*.*e*. maltase, amylase), lipolytic enzymes (*i*.*e*. lipase), and phosphatases (*i*.*e*. alkaline phosphatase) [11]. In carnivorous species, higher levels of proteolytic enzyme activity are generally reported as compared to herbivorous or omnivorous fish [12]. On the other hand, carbohydrases are not considered to be of primary importance in the digestive processes of predatory fish because this kind of enzymatic activity is quite limited as compared to the same activity measured in herbivorous and omnivorous fish [13]. As a whole, protease (*i*.*e*. trypsin) activity in carnivorous species increases with age, while it decreases in herbivorous fish. Carbohydrase (*i*.*e*. amylase) activity decreases with age in carnivorous species, while it increase in herbivorous and omnivorous fish [12].

Season and temperature are the most extrinsic factors influencing the metabolic rate of aquatic organisms. Most aquatic organisms are ectothermic animals. As a consequence, environmental temperature is a key factor controlling the basic physiological processes responsible for their survival, growth and reproduction [14]. The peculiarity of ectothermic animals lies in their ability to adapt to different temperatures in their environment, with maximum and minimum tolerance limits (tolerance range). Several studies have been conducted to determine the influence of water temperature during various seasons on fish digestive processes and enzyme activities [7, 14–17]. In the pyloric caecum of Japanese yellowtail, the activity of stomach proteolytic enzymes decreased in cold water, while enzyme activity in the intestine increased [16]. In agreement with these results, protease and lipase activity levels in the anterior section of the intestine tended to be higher in winter, likely due to slower gut motility at colder water temperatures [17].

Diverse comparative studies of digestive enzymes in different fish species have been reported [4, 18]. However, most studies have focused on digestive enzymes of economically valuable fish species.

The threespine stickleback (*Gasterosteus aculeatus*) is a noncommercial small-body teleost fish (35–55 mm, mean standard size) widely found in boreal and temperate regions of the northern hemisphere. It inhabits coastal marine waters, brackish waters, and a wide array of freshwater habitats [19]. The interest of stickleback is mainly ecological and scientific because it occupies an intermediate trophic level [20] and contributes to valuable ecosystem functions: it is considered as an omnivore which feeds on small invertebrates and different insect larvae, but can also serve as prey for many species of carnivorous benthic invertebrates (when juvenile), birds and fish (adults and older stages) [19, 20]. Thus, this species is generally considered as a scientific treasure. It is a well-studied model for experimental studies in multidisciplinary fields of biology: aquatic evolutionary biology, ecology and behaviour [21, 22]. It is also considered as a good sentinel fish species in aquatic ecotoxicology [23–25].

Several authors have addressed threespine stickleback digestive physiology [19, 26–29]. Their studies concerned the ingestion process (food type and availability, frequency of ingestion, foraging behaviour) and the resulting energetic aspect. However, to our knowledge, no study has focused on digestion or on the digestive enzymes of this animal model. We addressed the enzymatic aspect of *G aculeatus* digestion to supplement existing data on the digestive process and energy metabolism of this species, and for long term consider using these parameters as new biomarkers in ecotoxicology. Since temperature is the most determining factor of fish physiology (it influences survival, growth and reproduction), we studied its influence on digestive enzyme (amylase, intestinal alkaline phosphatase, trypsin) activity levels. To avoid or control for the potential influence of other parameters such as age, size, or food availability, we set up two original experimental designs under laboratory conditions using threespine stickebacks reared at the INERIS (French National Institute for Industrial Environment and Risks). First, stickebacks were exposed to a temperature-photoperiod cycle that mimicked seasonal (spring to autumn) variations for 240 days. For the second experiment, stickebacks were exposed to three steady-state temperatures (16, 18 and 21°C) for 120 days. Sticklebacks are typically present in waters with temperatures ranging from 4 to 20°C [19], so 16–18°C appeared to be an optimal temperature range for their growth and reproduction (Guderley 1994), while 21°C is in the range of stressful temperatures for them [30]. The temperatures we selected corresponded to a global rise of 1.8–4.0°C as predicted in a few decades by climate change scenarios [31]. We also studied gut morphometric parameters (RGM, RGL and ZI) in both experiments.

## Materials and methods

### Ethics statement

This experiment was conducted in accordance with the European directive 2010/63/UE for the protection of animals used for scientific purposes. The INERIS registration number, where the experiments were conducted, is the C60-769-02. The experimental protocols were submitted and reviewed by a French nationally recognized ethical committee: CREMEAP (Comité Régional d’Ethique en Matière d’Expérimentation Animale de Picardie), under the registration number 96.

### Fish origin and acclimation conditions

Our experiments were carried out on sticklebacks from the same population originating from INERIS artificial rivers (Verneuil-en-Halatte, France). Sticklebacks were all born the same year after natural reproduction in mesocosms between May and September and were a few (6–11) months old at the beginning of the experiment. Three months before the start of the experiments, sticklebacks were transferred for acclimation to experimental temperatures (14°C for experiment 1; 16°C for experiment 2) to 300-liter laboratory opaque tanks with a continuous freshwater circulation system (0 ppt), a photoperiod according to the experiment (see below) and a constant feeding regime with the following properties: 5% of crude protein, 0.15% of crude fat, 1.6% of crude fiber and 91.1% of moisture (frozen commercial chironomid larvae *ad libitum*, 3% of body weight/day, Ocean Nutrition^TM^, Belgium). Each tank was under continuous aeration to maintain a saturated oxygen concentration. Sticklebacks were periodically (every 15 days) classified according to their size classes, *i*.*e*. small (30–35 mm, 36–40 mm) and large (41–45 mm and >45 mm) and distributed among the different tanks. The feeding level was adjusted at each time-point and classification, and was maintained as a constant ratio of the fish mass, as described by Leloutre [32].

### Experimental design

#### Experiment 1: Influence of the temperature-photoperiod cycle

In the first experiment, after the acclimation period (see above), sticklebacks were exposed to a temperature cycle (14 to 20°C) and photoperiod cycle (Table 1). The temperature was increased regularly (1°C month^-1^ on average) during the first 180 days of the experiment, and then decreased until the end (1.5°C month^-1^ on average) to reach approximately 17°C on day 240. In addition, the photoperiod was also modulated (see Table 1).

**Table 1:** Water parameters and photoperiod during the mimicked seasonal variation experiment. Only temperatures and the photoperiod were modulated. The pH, dissolved oxygen and conductivity changed with water temperature, but their effects were not studied.

Conditions	Water parameters				Photoperiod (light/ dark h/h)
	Temperature (°C)	pH	Dissolved oxygen (mg/l)	Conductivity (μS)	
**Day 0**	14.29 ± 0.28	8.80 ± 0.17	8.04 ± 0.12	596.17	10/14
**Day 60**	16.13 ± 0.30	8.42 ± 0.15	7.96 ± 0.03	571.46	14/10
**Day 120**	18.32 ± 0.41	6.14 ± 0.64	7.65 ± 0.07	482.68	14/10
**Day 180**	20.02 ± 0.39	6.77 ± 0.35	7.93 ± 0.03	452.52	14/10
**Day 240**	17.24 ± 0.38	7.86 ± 0.79	8.34 ± 0.20	645.58	12/12

Temperature and photoperiod varied together as they do in natural environments, and their effects were not distinguishable. To assess their combined effects, we tested differences between measurement times. Moreover, we also studied the potential influence of sex and fish size. To describe enzyme and digestive tract parameters, we discretized fish length in four classes: 30–35 mm, 36–40 mm, 41–45 mm, and >45 mm.

Thus, according to the acclimation conditions, four tanks (one *per* size class) containing 300 individuals each were submitted to the temperature-photoperiod cycle. Every other month, 15 individuals were sampled in each tank. In order to minimize stress as much as possible, sampling was carried out in one go, and size was measured only after dissection, and then classification into the correct size group was performed. This explains why different numbers of sticklebacks were analyzed each time. Water parameters (dissolved oxygen, pH and temperature) were assessed throughout the experiment to ensure optimal water quality; their effects on digestive enzymes and gut morphometric parameters were not studied (Table 1). Individuals from the different tanks were fed similarly to acclimation conditions according to fish mass. Due to technical issues, 5 samples were lost on sampling days 180 and 240.

#### Experiment 2: Influence of prolonged maintenance of sticklebacks at three different water temperatures

In the second experiment, three water temperatures were selected: (i) 16°C and 18°C, representing optimal temperatures for sticklebacks [33], and (ii) a higher temperature of 21°C, in the range of stressful temperatures for sticklebacks [30]. To investigate the effect of temperature acclimation on stickleback digestive process, homogeneous reproductive adult sticklebacks (955.80 ± 55.13 mg and 40.40 ± 0.49 mm) were used. After a 3-month-long acclimation period at 16°C, sticklebacks were randomly transferred into three new 300-liter opaque tanks (n = 30/tank/condition), with constant feed (frozen commercial chironomid larvae *ad libitum*, with the same composition as for acclimation conditions) and photoperiod (14h/10h light/dark cycle). Water temperature was adjusted at a rate of 0.5°C day^-1^, using TANK^®^ water conditioner (TK500, TECO SRL, Italy) for 18 and 21°C. Once the desired temperatures were reached, sticklebacks were maintained there for 120 days, and ten individuals from each group were sampled on days 15, 60 and 120 of the experiment. During the experiment, water parameters (dissolved oxygen, pH and temperature) were continuously monitored (Table 2).

**Table 2:** Water parameters recorded during the prolonged maintenance of sticklebacks at 16, 18 and 21°C.

Conditions		Water parameters			
		Temperature (°C)	pH	Dissolved Oxygen (mg/l)	Conductivity (μS)
**Day 15**	**16°C**	16.09 ± 0.18	8.18 ± 0.13	9.25 ± 0.23	440.80 ± 12.76
	**18°C**	17.83 ± 0.71	8.20 ± 0.13	8.80 ± 0.47	459.33 ± 12.39
	**21°C**	19.98 ± 1.62	8.13 ± 0.12	8.28 ± 0.64	485.90 ± 29.03
**Day 60**	**16°C**	16.30 ± 1.06	7.66 ± 0.13	8.25 ± 0.34	375.76 ± 12.20
	**18°C**	18.25 ± 0.07	7.79 ± 0.08	8.25 ± 0.18	393.15 ± 9.74
	**21°C**	21.25 ± 0.09	7.65 ± 0.23	7.61 ± 0.32	422.30 ± 10.73
**Day 120**	**16°C**	16.11 ± 0.31	7.47 ± 0.17	8.59 ± 0.12	328.50 ± 53.77
	**18°C**	18.02 ± 0.15	7.59 ± 0.07	8.29 ± 0.11	345.00 ± 46.65
	**21°C**	20.97 ± 0.05	7.59 ± 0.05	8.15 ± 0.08	387.75 ± 69.97

### Enzyme analysis and biometric parameters

For all experiments and at each sampling date, a 24-hour starvation period was applied before stickleback euthanasia, as recommended by Debnath [4]. Sticklebacks were sacrificed by cervical dislocation after anesthesia with tricaine methanesulfonate MS222 (70 mg. L^-1^, SIGMA-ALDRICH, France), weighed, and then standard length was measured. The whole digestive tract was removed on ice, rinsed with cold Tris-HCl buffer (0.01 M, pH 7, SIGMA-ALDRICH, France), cleaned of exterior fat, weighed and measured. Samples were then homogenized with ceramic (3 mm Ø) and glass (1mm Ø) beads in cold Tris-HCl buffer (0.01 M, pH7), using PRECELLYS24^**®**^ homogenizer (BERTIN TECHNOLOGIES, France), at 5,500 rpm 2x10 sec, and centrifuged at 15,000 x g for 30 min at 4°C. Supernatants were stored at -80°C until analysis. Measurements of amylase and intestinal alkaline phosphatase (IAP) activity levels according to Junge et al. [34] and Panteghini and Bais [35], respectively, were performed with adapted methods, using Thermo-Scientific Gallery ready-to-use reagents. Trypsin activity measurements were performed according to the Garcia-Carreño and Haard [36] method, using N-benzoyl-DL-arginine 4-nitroanilide hydrochloride (BAPNA, 3 mM) as a substrate. All enzymatic assays were adapted on the Gallery™ Automated Photometric Analyzer (Thermo Fisher Scientific Oy) and performed at 37°C, by kinetic colorimetric assay at 405 nm. Results are reported in U. g^-1^ of gut tissue. dx.doi.org/10.17504/protocols.io.nmtdc6n [PROTOCOL DOI].

For both experiments, after dissection, sex and gonado-somatic indices (GSIs) = gonad mass (g) x [body mass (g)]^-1^ × 100, [37] were determined. Gut parameters were calculated, *i*.*e*. (i) relative gut mass (RGM) = gut mass (g) x [body mass (g)]^-1^, (ii) relative gut length (RGL) = gut length (mm) x [standard length (mm)]^-1^ according to German and Horn [8], and (iii) Zihler’s Index (ZI) = gut length (mm) x [10 x (body mass (g) 1/3)]^-1^ according to Zihler [9]. Fulton’s condition factor (K) = body mass (g) x [length (mm)^3^]^-1^ x 100 was also calculated according to Htun-Han [38].

### Statistical analysis

Data were processed using R statistical software (v3.3.1) at α = 0.05 and were analyzed using ANOVA or ANCOVA. Normality and homogeneity tests (Shapiro and Levene tests, respectively) were used. When normality or homoscedasticity were not met, data were transformed using Box-Cox, log, or square root method. Pair-wise t-test, with Bonferroni correction, was used as post hoc test to compare groups.

In experiment 1, firstly, digestive enzyme parameters (amylase, IAP and trypsin) and gut morphometric parameters (RGL, RGM, ZI) were analyzed at each time-point independently using ANCOVA. Fish length was used as a covariate and sex as a factor. To assess the combined effects of temperature and photoperiod, differences over time were investigated. Then, in a second step, one-way ANOVA was performed with time-point as a factor either on all sticklebacks together (no sex effect, fish length effect neglected) or on males and females taken separately (fish length effect neglected) according to the results of the first analysis. Biometric parameters (body mass, standard length, Fulton’s condition factor and GSI) were analyzed first at each time-point independently, using two-way ANOVA, with sex and size classes as factors. Since sex had no effect on these parameters, except for GSI, one-way ANOVA was performed for each size class, with time-point as a factor either on all sticklebacks together (body mass, standard length, Fulton’s condition factor) or on males and females taken separately (for GSI).

In experiment 2, we first analyzed the effect of sex on the different parameters at each time-point independently, using two-way ANOVA. Sex and temperature condition were considered as factors. Since no effect of sex was recorded for any of the parameters except GSI, two-way ANOVA was performed with temperature and time as factors. This analysis was conducted on all sticklebacks, except GSI that was performed on males and females taken separately, to assess differences over time.

Finally, principal component analysis (PCA) was performed on digestive enzyme activity levels for the two experiments and included the other parameters: fish biometric parameters (body mass, standard length, Fulton’s Factor and GSI), gut morphometric parameters (RML, RGM and ZI), and water parameters (dissolved oxygen, pH and temperature), as supplementary variables (explanatory variables).

## Results

### Experiment 1: Influence of the temperature-photoperiod cycle

First, the potential effect of sex on the different parameters was tested. No effect was observed (p<0.05) on most of the parameters except GSI (Table 3) and amylase activity at one time-point (Supplementary data, S1 Table and S1 Fig). In fact, amylase activity on day 60 was lower in females than in males (Supplementary data, S1 Fig). This difference was not observed at the other dates.

**Table 3:** Biometric and gut morphometric parameters measured in sticklebacks exposed to a temperature-photoperiod cycle after 0, 60, 120, 180 and 240 days.

Conditions		N	Biometric parameters					Gut morphometric parameters		
			Body mass (mg)	Standard length (mm)	Fulton’s Factor (K)	GSI		RGM	RGL	ZI
						Males	Females			
**Day 0 (14.29°C)**	**[30–35 mm]**	**15**	395.86 ± 69.10**a**	31.53 ± 1.35**a**	1.25 ± 0.09**a**	1.97 ± 1.73**a**	1.38 ± 0.81**a**	0.052 ± 0.005**a**	0.54 ± 0.06**a**	13.23 ± 1.97 **a**
	**[36–40 mm]**	**15**	688.73 ± 121.03**b**	37.13 ± 1.40**b**	1.34 ± 0.17**ab**	1.18 ± 0.48**a**	2.09 ± 0.53**a**	0.039 ± 0.005**b**	0.64 ± 0.09 **a**	10.64 ± 1.82 **b**
	**[41–45 mm]**	**13**	1200.15 ± 224.15**c**	43.38 ± 1.60**c**	1.46 ± 0.23**b**	0.95 ± 0.32**a**	2.00 ± 0.47**a**	0.045 ± 0.007**c**	0.62 ± 0.08 **a**	6.55 ± 1.58 **c**
	**[> 45 mm]**	**17**	1567.18 ± 241.10**d**	48.95 ± 2.88**d**	1.34 ± 0.10**b**	0.94 ± 1.03**a**	1.53 ± 1.15**a**	0.045 ± 0.007**c**	0.47 ± 0.22 **a**	4.67 ± 1.90 **d**
**Day 60** **(16.13°C)**	**[30–35 mm]**	**15**	481.86 ± 58.81**a**	33.06 ± 1.38**a**	1.32 ± 0.09**a**	0.95 ± 0.37**a**	1.03 ± 0.60**a**	0.047 ± 0.005**a**	0.62 ± 0.02 a*	13.02 ± 1.49 **a**
	**[36–40 mm]**	**4**	879.75 ± 164.76**b**	38.75 ± 0.95**b**	1.50 ± 0.19**a**	0.63 ± 0.46**a**	12.26**abc**	0.039 ± 0.008**b**	0.57 ± 0.06 **a**	7.77 ± 1.07 b***
	**[41–45 mm]**	**23**	1089.47 ± 200.14**c**	42.47 ± 1.78**c**	1.41 ± 0.19**a**	0.85 ± 0.35**a**	9.39 ± 8.79**bc**	0.038 ± 0.007**b**	0.62 ± 0.09 **a**	7.47 ± 2.11 **b**
	**[> 45 mm]**	**18**	1618.11 ± 346.09**d**	49.00 ± 2.86**d**	1.36 ± 0.19**a**	0.60 ± 0.44**a**	7.47 ± 7.44bc*	0.034 ± 0.005b*	0.57 ± 0.10 **a**	5.27 ± 1.22 **c**
**Day 120** **(18.32°C)**	**[30–35 mm]**	**13**	511.00 ± 81.61a*	33.15 ± 2.11**a**	1.40 ± 0.20a*	0.37 ± 0.34**a**	0.89 ± 0.29**a**	0.046 ± 0.008**a**	0.50 ± 0.10 **a**	10.13 ± 2.66 a***
	**[36–40 mm]**	**12**	777.75 ± 118.63**b**	37.75 ± 1.54**b**	1.43 ± 0.10**a**	0.68 ± 0.43**a**	4.14 ± 7.11 **a**	0.041 ± 0.006**a**	0.48 ± 0.08 a*	7.16 ± 1.40 b***
	**[41–45 mm]**	**22**	1163.27 ± 193.50**c**	43.40 ± 1.43**c**	1.41 ± 0.17**a**	0.86 ± 0.36**a**	8.10 ± 6.2**a**	0.045 ± 0.007**a**	0.58 ± 0.10 **a**	6.58 ± 1.08 **b**
	**[> 45 mm]**	**13**	1444.23 ± 178.76**d**	47.76 ± 2.04**d**	1.32 ± 0.10**a**	0.58 ± 0.004**a**	7.29 ± 9.46 **a**	0.039 ± 0.009**a**	0.53 ± 0.10 **a**	5.45 ± 1.41**c**
**Day 180 (20.02°C)**	**[36–40 mm]**	**15**	774.00 ± 103.93**a**	38.40 ± 1.24**a**	1.36 ± 0.14**a**	0.34 ± 0.19 **a**	2.23 ± 3.17**a**	0.050 ± 0.007**a**	0.61 ± 0.08 **a**	9.19 ± 1.30 **a**
	**[41–45 mm]**	**22**	1202.90 ± 176.76**b**	43.31 ± 1.52**b**	1.47 ± 0.16**a**	0.61 ± 0.39 a*	7.02 ± 6.04**a**	0.052 ± 0.009**a**	0.64 ± 0.06 **a**	7.10 ± 1.00 **b**
	**[> 45 mm]**	**7**	1556.57 ± 238.08**c**	47.00 ± 1.15**c**	1.49 ± 0.19**a**	0.82 ± 0.80**a**	7.29 ± 9.46**a**	0.049 ± 0.01**a**	0.64 ± 0.03 **a**	5.96 ± 0.87 **b**
**Day 240 (17.24°C)**	**[41–45 mm]**	**14**	1145.00 ± 146.37**a**	43.28 ± 1.72**a**	1.41 ± 0.16**a**	0.72 ± 0.28**a**	1.33 ± 1.36**a**	0.042 ± 0.007**a**	0.58 ± 0.05 **a**	6.74 ± 1.05 **b**
	**[> 45 mm]**	**12**	1524.16 ± 179.06**b**	47.25 ± 1.05**b**	1.44 ± 0.18**a**	1.11 ± 0.49**a**	1.03 ± 0.36**a**	0.040 ± 0.005**a**	0.57 ± 0.06 **a**	5.34 ± 0.72 **c**

Different letters indicate significant differences between size class groups of the same month (mean ± S.D. p<0.05). Asterisks indicate significant differences between fish of the same size class at different time conditions with respect to initial values of day 0 (*p<0.05; **p<0.001; ***p<0.00001). GSI: Gonado-Somatic Index; RGM: Relative Gut Mass. RGL: Relative Gut Length; ZI: Zihler’s Index.

Table 3 presents the different parameters according to size classes and time-points. As described above, only 15 sticklebacks *per* condition were caught at each sampling, and they were classified in the appropriate experimental condition after dissection; this is why groups are numerically unbalanced (Table 3). In addition, because sticklebacks belonged to the same generation, the smallest ones grew up and were no longer small enough from day 240 for the 35–40 mm size class and from day 180 for the 30–35 mm size-class. ANCOVA was used with length as a covariate (numerical continuous variable) to avoid this bias in statistical analyses.

**Fig 1:**
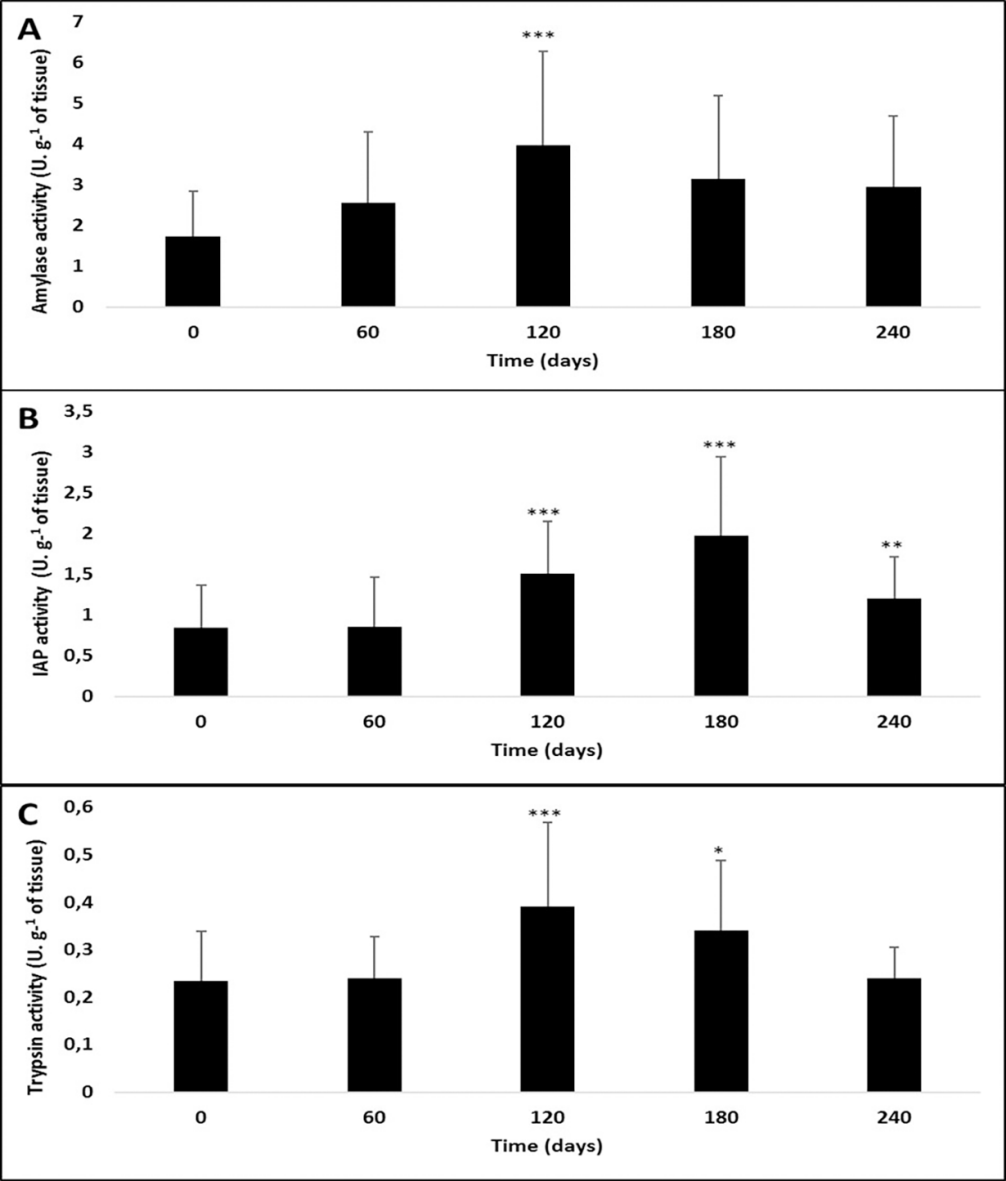
Effect of time condition on the activities of three digestive enzymes in *G*. *aculeatus*. **(A) Amylase, (B) Intestinal Alkaline Phosphatase (IAP), and (C) Trypsin.** Asterisks indicate significant differences in comparison with day 0 (*p<0.05; ***p<0.00001).

Throughout the experiment, amylase activity was the highest, followed by IAP activity, and then trypsin activity. No overall effect of size was observed (see supplementary data), except for amylase and trypsin in a few individual cases (Supplementary data, S1 Table and S1 and S3 Figs). Time condition had a significant effect on the activity of the three digestive enzymes (Fig 1). Overall, the activity levels of stickleback digestive enzymes increased significantly on days 120 (18.32°C, Pair-wise t-test: p< 0.00001) and 180 (20.02°C, Pair-wise t-test: p< 0.05 and decreased on day 240 (17.24°C), as compared to day 180. The lowest mean activity levels of the three digestive enzymes were recorded on day 0, while the highest values were observed on day 120 for amylase and trypsin, and on day 180 for IAP (Fig 1).

Concerning gut morphometric parameters (Table 3), RGL and RGM values ranged from 0.47 to 0.64 and from 0.034 to 0.052, respectively. Neither fish size nor time affected RGM or RGL, except in some cases: RGM had decreased while size had increased on day 60, and RGM had increased in 35–40 mm sticklebacks on day 240 as compared to day 0 (Supplementary data, S2 Table). On the other hand, Zihler’s index (ZI) was strongly affected by fish size (one-way ANOVA, p<0.0001) at each sampling date, with higher values for small fish, ranging from 5.34 on day 240 (> 45 mm) to 13.23 on day 0 (30–35 mm).

For each size class, no overall difference in stickleback body mass or length was observed throughout the experiment. As expected, there was a significant increase in body mass and length according to size class at each date. Fulton’s condition factor (K) was the same for all size groups. As regards GSI, despite an increase in absolute values from day 60 to day 180 indicating the onset of reproduction in a few individuals (especially females), no overall significant differences were observed between the different size classes or between sampling dates due to the high variability recorded within groups.

**Fig 2:**
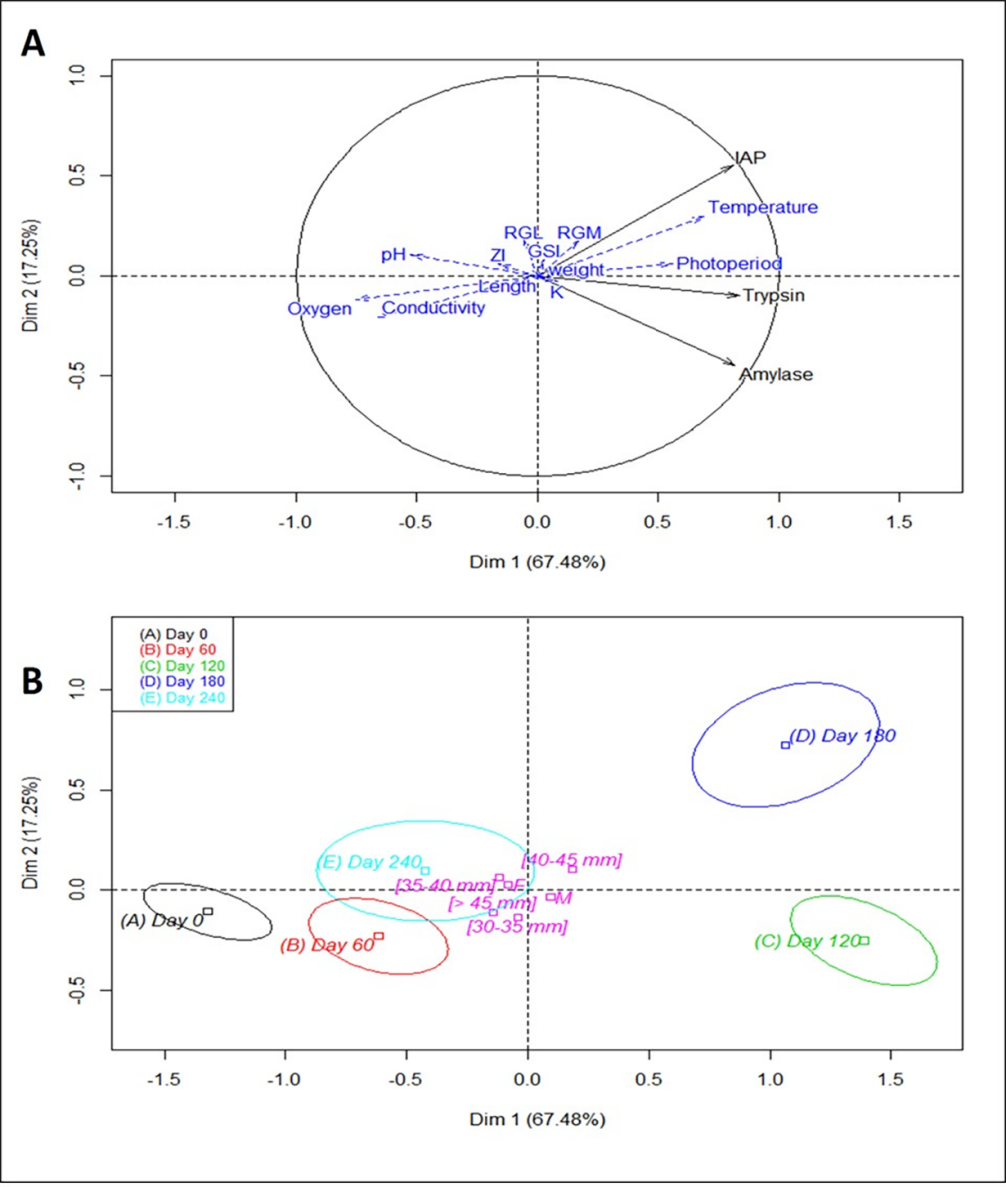
PCA model of digestive enzyme activities measured in experiment 1, in contrast with quantitative variables (Fig 2A): fish biometric parameters (weight, length, K: Fulton’s Condition Factor, GSI: Gonado-somatic index), gut morphometric parameters (RGM: Relative Gut Mass, RGL: Relative Gut Length, ZI: Zihler’s Index), water parameters (temperature, conductivity, dissolved oxygen, pH), and time as qualitative variables (Fig 2B).

A PCA was conducted including the following explanatory variables: water parameters, photoperiod, biometric parameters, and gut morphometric parameters (Fig 2A). Individuals were distinguished by the first axis according to time conditions (Fig 2B) and were explained positively by water temperature and photoperiod (R^2^ = 69% and 59% respectively, p<0.00001), and negatively by oxygen, conductivity and pH (R^2^ = 75%, 66% and 53% respectively, p<0.00001). None of the biometric and gut morphometric parameters explained the variance of the three digestive enzyme activities.

### Experiment 2: Influence of prolonged maintenance of sticklebacks at three different water temperatures

This experiment started with sticklebacks of the same size and weight in order to evaluate the effect of three temperatures and durations on their digestive capacity. As in the first experiment, there was no effect of sex on biological parameters except for GSI.

**Fig 3:**
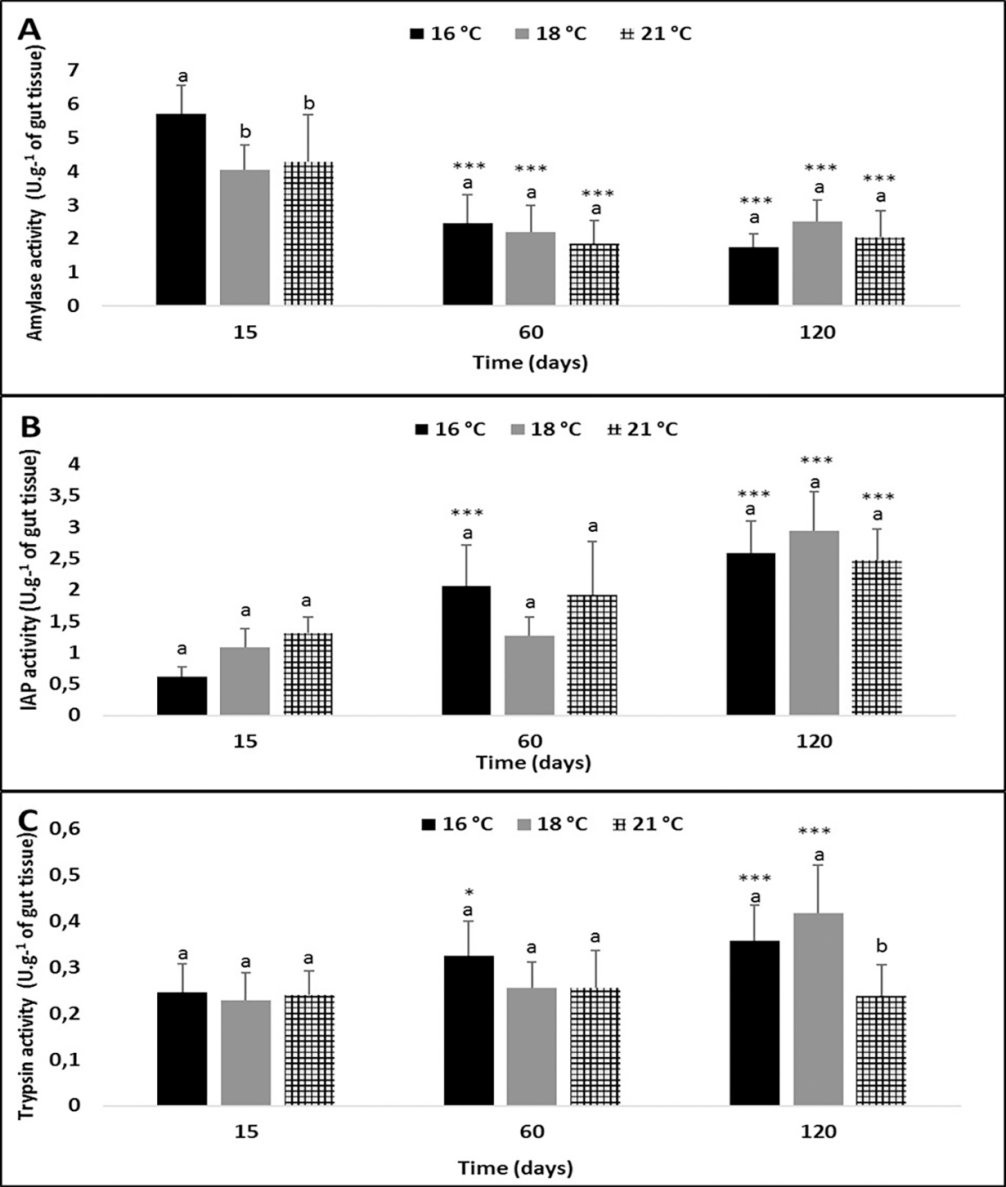
Effect of water temperature and time on the activity of three digestive enzymes in *G*. *aculeatus*. **(A) Amylase, (B) Intestinal Alkaline Phosphatase (IAP), and (C) Trypsin.** Different letters indicate significant differences between temperature groups of the same time condition (mean ± S.D; n = 10, P<0.05). Asterisks indicate significant differences with respect to initial values measured on day 15 (*p<0.05; **p<0.001; ***p<0.00001).

Duration was the most impacting factor on the activity of the three digestive enzymes (Two-way ANOVA p< 0.00001, Fig 3). Similarly, to the first experiment, amylase activity was highest, followed by IAP activity and trypsin activity, respectively.

Overall, the highest level of amylase activity was recorded on day 15 in all groups and decreased significantly from day 60 to day 120 (Pair-wise t-test, p< 0.00001). Trypsin and IAP activity levels increased significantly from day 60 at 16°C (Pair-wise t-test, 0.02 and p< 0.00001 for trypsin and IAP, respectively), and only on day 120 at 18°C (Pair-wise t-test, p<0.00001) as compared to day 15. Amylase activity was negatively correlated to weight and length (R^2^ = 53 and 55%, respectively). Trypsin and IAP activity levels had significantly increased on day 120 and were positively correlated to weight (R^2^ = 55 and 73%, respectively, p< 0.0001) and length (R^2^ = 51 and 68%, respectively, p< 0.0001), except at 21°C. At that temperature, trypsin activity remained the same throughout the experiment and was lower than activity at the other water temperatures (16 and 18°C) on day 120.

Temperature influenced amylase activity only on day 15, with higher activity at 16°C than in the other groups (Pair-wise t-test, P<0.0001). In the highest-temperature group (21°C) trypsin activity was lowest on day 120 (Pair-wise t-test, p<0.001) as compared to the other groups. When sticklebacks were exposed to the highest water temperature (21°C), a significant increase in IAP activity was observed on day 120 (Pair-wise t-test, p<0.00001). However, trypsin activity remained stable throughout the experiment, and similar to the activity measured on day 15.

Morphometric parameters (Table 4) were mainly affected by duration. Only RGL was not affected by time or temperature and ranged from 0.59 to 0.66. RGM ranged between 0.032 and 0.048 and had significantly increased on day 120 (Pair-wise t-test, p <0.05), while ZI significantly decreased in all groups from day 60 (Pair-wise t-test, p< 0.001) as compared to day 15, and ranged from 4.91 to 7.83.

**Table 4:** Biometric and gut morphometric parameters measured in fish exposed to three fixed water temperatures (16, 18 and 21°C) for 120 days.

Conditions		N	Biometric parameters					Gut morphometric parameters		
			Body mass (mg)	Standard length (mm)	Fulton’s Factor (K)	Gonado-Somatic index		RGM	RGL	ZI
						Males	Females			
**Day 15**	**16°C**	**10**	1124.53 ± 130.44**a**	42.00 ± 1.25**a**	1.51 ± 0.12**a**	1.34 ± 0.45**a**	5.75 ± 2.95**a**	0.034 ± 0.003**a**	0.59 ± 0.06**a**	6.67 ± 0.89**ab**
	**18°C**	**10**	1029.46 ± 184.18**a**	41.80 ± 2.11**a**	1.40 ± 0.13**a**	1.13 ± 0.30**a**	4.71 ± 3.50**a**	0.035 ± 0.006**a**	0.60 ± 0.06**a**	7.53 ± 1.11**bc**
	**21°C**	**10**	966.26 ± 127.71 **a**	40.73 ± 1.79**a**	1.42 ± 0.08**a**	1.27 ± 0.27**a**	7.63 ± 2.71**a**	0.037 ± 0.005**a**	0.61 ± 0.09**a**	7.83 ± 0.97**c**
**Day 60**	**16°C**	**10**	1699.33 ± 310.70a***	46.06 ± 1.94a***	1.73 ± 0.26**a**	0.83 ± 0.18a*	8.81 ± 3.85**a**	0.033 ± 0.007**a**	0.60 ± 0.07**a**	5.03 ± 0.72a***
	**18°C**	**10**	1410.00 ± 246.19ab***	44.22 ± 2.33**a**	1.62 ± 0.19**a**	0.96 ± 0.23**a**	10.71 ± 5.62a*	0.032 ± 0.008**a**	0.59 ± 0.07**a**	5.67 ± 0.97a***
	**21°C**	**10**	1240.00 ± 204.38b**	43.33 ± 2.66a***	1.51 ± 0.12**a**	0.79 ± 0.21**a**	9.35 ± 3.16**a**	0.036 ± 0.006**a**	0.55 ± 0.11**a**	5.78 ± 0.94a***
**Day 120**	**16°C**	**10**	1844.53 ± 269.89a***	48.66 ± 2.71a***	1.60 ± 0.18**a**	1.00 ± 0.42**a**	2.02 ± 2.14**a**	0.044 ± 0.004a*	0.61 ± 0.07**a**	4.93 ± 0.64a***
	**18°C**	**10**	2066.00 ± 293.06a***	50.20 ± 2.70a***	1.63 ± 0.16a*	0.84 ± 0.27**a**	2.59 ± 1.72**a**	0.045 ± 0.007a**	0.66 ± 0.04**a**	4.91 ± 0.37a***
	**21°C**	**10**	1431.21 ± 277.21b***	45.73 ± 3.84b***	1.41 ± 0.41**a**	0.42 ± 0.18b***	1.32 ± 0.45a*	0.048 ± 0.009a**	0.59 ± 0.05**a**	5.93 ± 1.11**a**

Different letters indicate significant differences between temperature groups at the same time condition (mean ± S.D; n = 10. p<0.05). Asterisks indicate significant differences with respect to initial values measured on day 15 (*p<0.05; **p<0.001; ***p<0.00001). GSI: Gonado-Somatic Index; RGM: Relative Gut Mass. RGL: Relative Gut Length; ZI: Zihler’s Index.

Sticklebacks grew significantly in body mass and body size throughout the experiment. Those from the 21°C group exhibited the significantly lowest body mass after 60 days and the lowest body size after 120 days as compared to the other groups. Overall, neither temperature nor duration affected Fulton’s condition factor (K) or GSI in both males and females, except in a few exceptional cases (Table 4).

**Fig 4:**
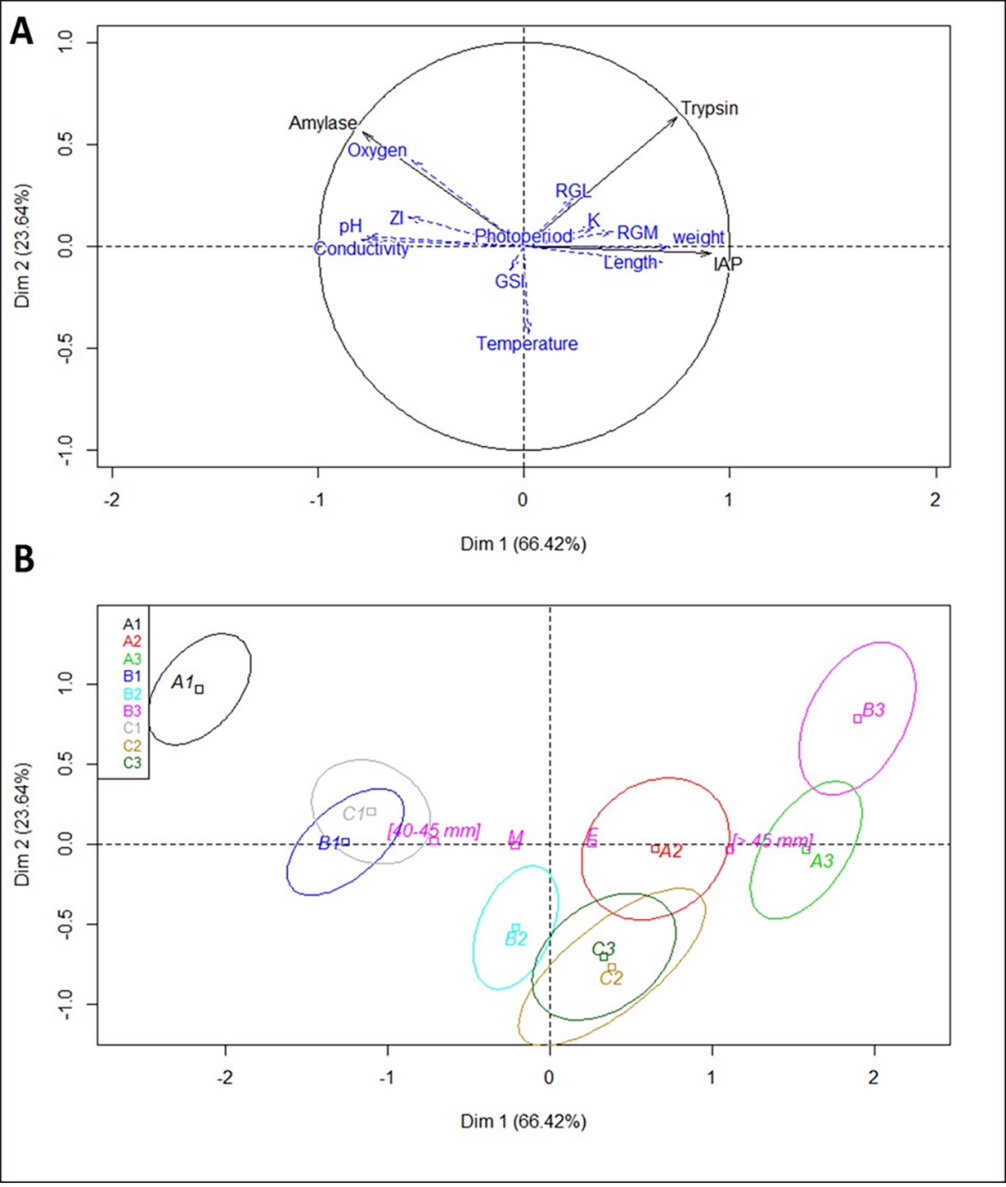
PCA model of digestive enzyme activities measured in experiment 2, in contrast with quantitative variables (Fig 4A): fish biometric parameters (weight, length, K: Fulton’s Condition Factor, GSI: Gonado-somatic index), gut morphometric parameters (RGM: Relative Gut Mass, RGL: Relative Gut Length, ZI: Zihler’s Index), water parameters (temperature, conductivity, dissolved oxygen, pH), and time as qualitative variables (Fig 4B). (IAP: Intestinal Alkaline Phosphatase. In Fig 4B, capital letters A, B and C represent the experimental water temperatures of 16, 18, 21°C, respectively. 1, 2 and 3 represent the experimental time periods of 15, 60 and 120 days, respectively.

A PCA was conducted on the activity of the three digestive enzymes, including the other parameters as explanatory factors (Fig 4A). Individuals were distinguished according to time conditions (Fig 4B). Data variance was explained by fish biometric parameters, with positive correlations with the first axis: weight (R^2^ = 71%, p< 0.00001) and length (R^2^ = 67%, p< 0.00001), by gut morphometric parameters: ZI (negative correlation, R^2^ = 56%, p< 0.00001) and RGM (positive correlation, R^2^ = 43%, p<0.00001), and by water parameters with a negative correlation: conductivity (R^2^ = 78%, p<0.00001), pH (R^2^ = 76%, p< 0.00001) and oxygen (R^2^ = 54%, p< 0.00001).

## Discussion

### Digestive characteristics of *G*. *aculeatus*

In this study, we focused on the activity of three digestive enzymes (*i*.*e*. amylase, trypsin and IAP) and three gut morphometric parameters (*i*.*e*. RGL, RGM and ZI) of the threespine stickleback. Sticklebacks were fed with a constant-composition food (frozen chironomid larvae). Under these fixed nutritional conditions, the activity of amylase was higher than that of trypsin, and this for all experiments. Higher amylase activity was reported in benthophage fish (bream, carp, roach) that usually feed on chironomid larvae than in other fish species (pike, burbot, perch) [39]. A higher amylase activity levels were generally noted in omnivorous fish [13]. In natural environments, sticklebacks have a wide choice of food items, and they hunt for prey visually. Thus, this species is admittedly omnivorous, with a preference for food of animal origin [29]. In literature, feeding habits of *G*. *aculeatus* were usually addressed by studying their stomach contents [29]. To our knowledge, this is the first study that addresses the digestive enzymes of *G*. *aculeatus*, and our first results concord with what was reported in literature on the feeding habits of this species, despite the lack of dietary diversity.

Concerning gut morphometric parameters, we described for the first time three parameters in relation to *G*. *aculeatus* gut morphology (RGL, RGM and ZI). RGL and ZI are crude measures and were explored as potential indices to identify feeding habits of fish based on their gut length [8]. According to feeding guilds reported by Al-Hussaini [10, 40], fish are recognized as carnivores, omnivores or herbivores, when RGL ranges respectively between (0.6–2.4), (1.3–4.2) and (3.7–6.0). According to ZI values, Kramer and Bryant [41] also categorized fish with a low body mass (0.3–3.0 g) as carnivorous (ZI = 2.3–3.2), omnivorous (ZI = 2.4–5.8) or herbivorous (ZI = 11.6–55.0). In our study, RGL ranged from 0.50 to 0.64 and corresponded to RGL values of carnivorous fish, and ZI values ranged between 4.91 and 13.60, and did not match with any of the three categories described above. Contrary to what has been reported in the literature for other species, the use of these parameters to classify sticklebacks according to their feeding habits is not obvious, which is probably due to the experimental conditions used in our study (*i*.*e*. calibrated and undiversified diet). In addition, sticklebacks have an elongated-fusiform body shape [28]. As reported by German and Horn [8], gut morphometric parameters should be treated with caution in fish with elongate, eel-like body shape such as pricklebacks.

RGM is usually addressed to evaluate the amount of tissue dedicated by fish to their digestive tract [8]. In our study, RGM values were higher in small sticklebacks (30–35 mm) than in large ones (> 45 mm) and suggest that small sticklebacks fed on chironomid larvae increased their gut mass to maximize extraction of nutrients and energy from their diet and ensure growth. Increasing gut mass is one of the mechanisms used by animals to increase energy intake from food [42].

This study provides new information to supplement existing data on the digestive process and energy metabolism of the threespine stickleback. However, the specific experimental conditions of this study (calibrated undiversified food ration) must be kept in mind when considering these first results. Further experiments are nedded with different diet to confirm these informations.

Substrate composition of diet is well known to be a modulating factor on digestive enzyme activities and gut morphometric parameters. Hence, in this study, we chose to control this parameter in order to study the effects of other factors such as fish size, sex and temperature.

### Effect of temperature on digestive capacity

The effect of temperature on the digestive capacity of *G*. *aculeatus* was addressed using two different experiments: *(i)* after exposure to temporal modulation of temperature and photoperiod miming seasonal variations and *(ii)* after long period exposure to three fixed temperatures. The activity of digestive enzymes was modulated in different ways depending on the exposure scenario. The effects of temperature on digestive enzymes can be direct [43], as they can be indirect via the modulation of food intake [44], or the gut morphometric parameters [42]. In our study, morphometric parameters were not affected by temperature in both experiments. Hence, digestive enzyme modulation could be the result of a direct effect of temperature by modulating synthesis and/or secretion process or indirect by altering the ingestion capacity of sticklebacks. Unfortunately, in our study food intake was not measured, and should be taken into account in further experiments. However, whether the effects are direct or indirect, in this study we do not address the mechanistic of thermal effect on the digestive enzymes. In fact, we wanted to determine how these parameters could react in front of temperature modulation and what if we could use these parameters as markers of a stressful situation such as a long exposure to a higher water temperature.

The first experiment showed temporal variation of digestive enzyme activity levels, with increases on days 120 (18.3°C) and 180 (20.0°C) when the water temperature and photoperiod were highest. Fish digestive enzyme activity can be affected by several factors, *e*.*g*. seasonal factors like temperature and the photoperiod [39, 45]. Seasonal variation of amylase and proteolytic activity levels was found in roach and rudd [7, 15]. Adaptation of enzyme activity levels to temperature was species- and/or food-dependent, so the author concluded that the two species had different strategies for seasonal adaptation of digestive enzymes that both achieved the same aim: in roach by direct temperature dependence, in rudd by inflexible annual rhythm (*i*.*e*. photoperiod), coinciding with the annual temperature pattern of the water. Carbohydrase activity levels increased in two teleost fish (bream and roach) when temperature rose to 20°C [46]. The author explained this elevation by a greater food intake coinciding with the higher temperature. In fact, increased food intake is one of the mechanisms aimed at offsetting increased energy demand. This increase generally results in greater digestive enzyme activity [42]. In natural conditions, a slight decrease in the amount of food eaten by sticklebacks was recorded during cold periods, followed by an increase during warmer periods, in relation to the increased energy demands during the breeding season [19, 28]; this supports our finding. Amylolytic enzyme activity increased to maximum levels in bream and roach during the warmer season, which coincides with the period of sexual maturity [12]. We evaluated reproduction based on GSI and showed no correlation with the activity levels of the three digestive enzymes, despite an increase of global GSI means from day 60 to day 180 that coincided with an increase in water temperature. This indicates that reproduction was set on in a few individuals (especially females).

The photoperiod and water parameters were the most explanatory variables for digestive enzyme activities. Temperature variations were associated with fluctuations in other water parameters (pH, dissolved oxygen and conductivity), so that their effects on digestive enzymes were not considered. Further experiments should explain their contribution. In the first experiment, the photoperiod was modulated along with water temperature and showed a positive correlation with the three digestive enzymes. Sticklebacks are visual predators, so their feeding behavior was affected by the day/night cycle, and it had either slowed down or completely stopped in nocturnal periods [47]. In miiuy croaker (*Miichthys miiuy*) larvae and juveniles, the photoperiod had no effect on trypsin or amylase activity levels, yet lipase activity was significantly higher in longer light/dark (18/6 and 24/0) photoperiods [45].

In the second experiment, there was no difference in digestive enzyme activity levels between sticklebacks submitted to the three temperatures, except for amylase on day 15 in the 16°C group and trypsin on day 120 in the 21°C group, and the adaptation patterns did not match with what was recorded in the first experiment when sticklebacks were exposed to a mimicked seasonal variation of temperature. In fish, temperature adaptation of enzymes may be achieved through several mechanisms: (a) changing the molecular conformation, (b) modulating energy activation, (c) changing affinity for the substrate, (d) modulating enzyme secretion, (e) producing various isoenzymes [43]. The absence of differences in digestive enzyme activity in sticklebacks from the different temperature groups can be explained by a prior adaptation of their digestive system to an increase in water temperature that was established before the start of the second experiment through production of isoenzymes with a large temperature range. When the second experiment began, sticklebacks were already at 16°C and were exposed to a progressive increase in temperature from day 0 (the beginning of experiment 1). Under a low rate of water temperature increase (0.04°C h^-1^) corresponding to the increase applied in the present study, amylolytic activity levels in goldfish, carp, roach and perch juveniles seem to be at the same levels in all seasons [48]. The temporal decrease of amylase activity levels in our three temperature groups could be explained by the production of new isoenzymes with low-energy activation, which are more efficient even at low concentrations. Lower-energy activation of amylase activity has been reported in different fish species as an adaptation of the digestive system to temperature maintenance through the production of new efficient isoenzymes [39].

Exposure of sticklebacks to three different temperatures for 120 days influenced their growth (weight and length), which was correlated to digestive enzyme activity (Fig 4). Environmental temperature has major effects on metabolism, growth, and fundamental biochemical processes, and these effects are well documented in the literature [47, 49]. In the early growth stages of almost all fish species, as well as in sticklebacks, growth rapidly increases when temperature rises, reaches a peak at optimum temperatures, and rapidly decreases when temperatures become adverse. We evidenced a limitation effect of the “high” 21°C water temperature on stickleback growth. Sticklebacks reared at the “high” temperature weighed less than those raised at 16 or 18°C. Several studies have addressed the effect of temperature on stickleback growth [19]. Sticklebacks can tolerate a wide range of temperatures but prefer relatively cool water (< 18°C) [33], which is in accordance with our results. However, the bioenergetics model of Hovel [49] suggested that 22°C could be the optimal temperature for growth with an upper limit of 25°C. But the model was designed based on a 3-day experimental design and did not address prolonged temperature exposure of sticklebacks.

Digestive capacity (e.g. proteolytic enzymes) and metabolic capacity required to support tissue protein synthesis have been reported as factors that can partly contribute to setting the rate of fish growth [50]. Some authors reported a correlation between growth and trypsin, chymotrypsin and alkaline phosphatase activity levels in fish [51, 52]. In the second experiment, trypsin activity did not change in sticklebacks exposed to 21°C, and was significantly lower than in sticklebacks from the 16 and 18°C groups on day 120. This low trypsin activity level may explain the difference in mean weights between sticklebacks from the 21°C group and sticklebacks from the other two groups. This difference can also be explained by the effect of temperature on stickleback metabolism. In fact, high temperature has been reported to increase the metabolic rate of living organisms [19]. In such a situation, optimum energy allocation is affected, and the energetic balance is redirected to maintenance rather than growth [53]. Decreased growth could be the consequence of an active metabolism following ingestion of a given amount of food [54]. A water temperature of 21°C corresponds to a predicted summer temperature reported by the IPCC [31] in a few decades. Our results show that exposure of sticklebacks to these elevated temperatures for a long period could be compromising for their physiology by affecting growth, which may subsequently have long-term impacts on other physiological functions such as reproduction. Sticklebacks (fed *ad-libitum*) probably allocated the energy from digested food to maximizing maintenance and reproduction while compromising growth and trypsin synthesis. This interesting result suggests that trypsin activity could be a potential marker of a thermal stress situation but requires further investigation to confirm it.

Water temperature had no major effect on gut parameters in both experiments, except on ZI in small fish (< 40 mm) which significantly decreased with the increase in stickleback body mass on days 60 and 120 as compared to day 0. Several studies have addressed the effect of temperature on the digestive tract of vertebrates, and have reported a decrease in intestinal length and mass when animals were exposed to elevated temperatures [55, 56]. This involution at warm temperatures could result from a regulatory mechanism that maintains the gut in an appropriate condition without incurring excessive energetic costs. Digestive tissues are among the most costly tissues to maintain in terms of energy. Therefore the intestine should be long enough for dietary nutrient uptake to be sufficient [57].

### Effect of body size on digestive capacity

Stickleback size had no or only a slight effect on the activity levels of the three digestive enzymes. This could be due to the way results were expressed (activity *per* gram of gut), suggesting that this allometric scaling already took into account the potential effect of size. Amylase and trypsin activity levels decreased with increasing age in European sea bass larvae [58]. These results were explained by the increase in total proteins in elderly larvae. To avoid the bias of the protein load, we chose to express enzymatic activity levels in units *per* gram of gut. Several studies have shown that fish age or developmental stage had variable effects on digestive enzymes depending on species and food habits, but most of them focused on early larval or juvenile stages of economically relevant fish species [12, 45, 59, 60]. Alkaline phosphatase activity increased with size in three teleost fish species (pike, perch, and roach). In contrast, carbohydrase (*i*.*e*. amylase) activity decreased with fish size [12]. German [61] compared digestive enzyme activity levels in four species of herbivorous and carnivorous pricklebacks (*Cebidichthys violaceus*, *Xiphister mucous*, *Xiphister atropurpureus* and *Anoplarchus purpurescens*) and determined the effects of age on these parameters. The author reported a size effect on certain digestive enzymes in some species but not in others. For example, in *Cebidichthys violaceus*, pepsin activity decreased significantly with a size increase, whereas in the other three species no significant difference was observed. Amylase activity increased in *Cebidichthys violaceus*, *Xiphister mucous* and *Xiphister atropurpureus* along with a size increase, whereas no changes were observed in *Anoplarchus purpurescens*. No size changes in relation to trypsin activity were observed in any of the four species. No effect of age or size on carbohydrase (*i*.*e*. amylase, sucrase, maltase) and lipase activity levels in Eurasian perch was noted either [62]. The authors discussed the results by specifying the restricted range of age classes covered by their study.

We investigated the effect of stickleback size on their digestive capacity in the first experiment. No size effect was recorded on RGM or RGL during the experiment. However, ZI was substantially impacted by size, and was higher in small sticklebacks (< 40 mm) than in large ones (> 40 mm). These differences were mostly related to body weight variations. The literature says that gut morphometric parameters increase with fish body size [8], but this is not in agreement with our results. These findings are not surprising considering the fusiform body shape of sticklebacks: the structure of sticklebacks’ intestine (a straight tube) is defined by its elongate body shape and cannot be longer than its abdominal cavity, unlike a flat and streamlined body shape which allows looping and convolution of the intestine [63].

## Conclusions

To our knowledge, this study is the first to characterize the digestive activity of *G*. *aculeatus* by focusing on three digestive enzymes (amylase, IAP, and trypsin) and three gut morphometric parameters (RGM, RGL, and ZI). Sticklebacks were fed exclusively with frozen chironomid larvae, with constant protein, fat and fiber composition. We chose to control the diet parameter, in order to study the effect of other factors (*i*.*e*. size, sex and temperature) on the digestive parameters. In these fixed nutritional conditions, sticklebacks exhibited higher amylase than trypsin activity in both experiments, characterizing an omnivorous fish, which is in accordance to feeding habits of this species, defined by others parameters (stomach contents) in the literature. When considering gut morphometric parameters, RGL and ZI failed to categorize sticklebacks according to their feeding habits, probably due to the lack of dietary diversity.

Our study showed no size effect, but a temporal variation of the three digestive enzymes was observed when temperature was progressively modulated to mimic seasonal variation. The activity of the three digestive enzymes was higher in hot periods (with a long photoperiod) and lower in cold days (with a short photoperiod). The highest levels of amylase and trypsin activity were observed at 18°C, while the highest IAP activity level was recorded at 20°C. When sticklebacks were exposed to three constant temperatures (16, 18, or 21°C), no differences were observed among groups, but a significant temporal effect was observed, with inverse evolution of the patterns between amylase activity and the activity of the other two digestive enzymes. The temporal effect on digestive enzymes was correlated with the effect of temperature on fish growth. Cool temperatures (16 and 18°C) favored a high growth rate (based on body mass evolution), while a temperature of 21°C limited growth efficiency even with a daily *ad libitum* diet. The results of this study suggest that in the context of global warming long exposure to a high water temperature (21°C) could compromise stickleback physiology by affecting their growth. Altered growth parameters (weight and size) were correlated to a decrease in trypsin activity and suggest that this enzyme could be used as a marker of thermal stress in the threespine stickleback.

While keeping in mind the specific experimental conditions of this study (calibrated undiversified food ration), these findings can be used to supplement existing data on the digestive process and energy metabolism of threespine sticklebacks. The absence of body size effects on digestive enzyme activity and the response of digestive enzymes to temperature changes can be considered as interesting results for possible use in an ecotoxicological context.

## Supporting information

S1 Table ANCOVA results of digestive enzymes activities in sticklebacks exposed at 0, 60, 120, 180 and 240 days to a temperature-photoperiod cycle. The model was constructed, considering size as continuous covariate, and sex as factor.
(DOCX)

S2 Table ANCOVA results of gut morphometric parameters in sticklebacks exposed at 0, 60, 120, 180 and 240 days to a temperature-photoperiod cycle. The model was constructed, considering size as continuous covariate, and sex as factor.
(DOCX)

S1 Fig Linear regression model explaining the effect of size and sex on amylase activity in sticklebacks exposed at 0, 60, 120, 180 and 240 days to a temperature-photoperiod cycle. The model was constructed, considering size as continuous covariate, and sex as factor. Note that a single regression line was plotted in absence of sex effect. Males were plotted in blue, and females in red.
(TIF)

S2 Fig Linear regression model explaining the effect of size and sex on the intestinal phosphatase alkaline activity (IAP) in sticklebacks exposed at 0, 60, 120, 180 and 240 days to a temperature-photoperiod cycle. The model was constructed, considering size as continuous covariate, and sex as factor. Note that a single regression line was plotted in absence of sex effect. Males were plotted in blue, and females in red.
(TIF)

S3 Fig Linear regression model explaining the effect of size and sex on trypsin activity in sticklebacks exposed at 0, 60, 120, 180 and 240 days to a temperature-photoperiod cycle. The model was constructed, considering size as continuous covariate, and sex as factor. Note that a single regression line was plotted in absence of sex effect. Males were plotted in blue, and females in red.
(TIF)
