# Supplementary material for: Digestive enzymes and gut morphometric parameters of threespine stickleback (Gasterosteus aculeatus): Influence of body size and temperature
Source: PLoS One. 2018 Apr 3;13(4):e0194932. doi: 10.1371/journal.pone.0194932 (PMC5882091; doi:10.1371/journal.pone.0194932)
Supplement: S1 Table — The model was constructed, considering size as continuous covariate, and sex as factor. (DOCX) [file pone.0194932.s001.docx]

# Supporting information

**S1 Table. ANCOVA results of digestive enzymes activities in sticklebacks exposed at 0, 60, 120, 180 and 240 days to a temperature-photoperiod cycle. The model was constructed, considering size as continuous covariate, and sex as factor.**

| Time condition | Covariate and factor | Amylase activity | | | | IAP activity | | | | Trypsin activity | | | |
| --- | --- | --- | --- | --- | --- | --- | --- | --- | --- | --- | --- | --- | --- |
|  |  | d.f. | SS | *F* | *p* | d.f. | SS | *F* | *p* | d.f. | SS | *F* | *p* |
| Day 0 | Size | 1 | 1.420 | 1.133 | 0.293 | 1 | 0.438 | 1.547 | 0.220 | 1 | 0.092 | 9.610 | **0.003^**^** |
|  | Sex | 1 | 0.300 | 0.242 | 0.625 | 1 | 0.001 | 0.005 | 0.944 | 1 | 0.001 | 0.112 | 0.739 |
|  | Size:Sex | 1 | 2.970 | 2.379 | 0.130 | 1 | 0.045 | 0.158 | 0.693 | 1 | 0.0003 | 0.035 | 0.853 |
| Day 60 | Size | 1 | 22.98 | 10.96 | **0.001^**^** | 1 | 0.087 | 0.234 | 0.631 | 1 | 0.029 | 4.287 | **0.043^*^** |
|  | Sex | 1 | 19.72 | 9.409 | **0.003^**^** | 1 | 0.022 | 0.059 | 0.808 | 1 | 0.0001 | 0.009 | 0.924 |
|  | Size:Sex | 1 | 17.82 | 8.500 | **0.005^**^** | 1 | 0.731 | 1.976 | 0.165 | 1 | 0.025 | 3.730 | 0.058 |
| Day 120 | Size | 1 | 22.02 | 4.405 | **0.042^*^** | 1 | 0.036 | 0.083 | 0.775 | 1 | 0.186 | 6.537 | **0.014^*^** |
|  | Sex | 1 | 4.070 | 0.814 | 0.372 | 1 | 0.189 | 0.438 | 0.512 | 1 | 0.003 | 0.133 | 0.717 |
|  | Size:Sex | 1 | 1.190 | 0.239 | 0.627 | 1 | 0.072 | 0.167 | 0.685 | 1 | 0.000 | 0.000 | 0.997 |
| Day 180 | Size | 1 | 1.130 | 0.281 | 0.599 | 1 | 0.152 | 0.168 | 0.684 | 1 | 0.006 | 0.268 | 0.608 |
|  | Sex | 1 | 15.98 | 3.976 | 0.054 | 1 | 3.165 | 3.512 | 0.0693 | 1 | 0.002 | 0.105 | 0.748 |
|  | Size:Sex | 1 | 2.170 | 0.541 | 0.467 | 1 | 1.212 | 1.345 | 0.254 | 1 | 0.004 | 0.188 | 0.667 |
| Day 240 | Size | 1 | 1.190 | 0.367 | 0.551 | 1 | 0.681 | 2.961 | 0.099 | 1 | 0.003 | 0.878 | 0.359 |
|  | Sex | 1 | 0.000 | 0.000 | 0.986 | 1 | 0.733 | 3.187 | 0.088 | 1 | 0.006 | 1.515 | 0.232 |
|  | Size:Sex | 1 | 1.750 | 0.538 | 0.471 | 1 | 0.041 | 0.180 | 0.675 | 1 | 0.001 | 0.400 | 0.534 |

d.f.: Degrees of Freedom; SS: Sums of Squares; *F*: ANCOVA F test; *p*: *p*-value; IAP: Intestinal Alkaline Phosphatase.
