# Supplementary material for: Digestive enzymes and gut morphometric parameters of threespine stickleback (Gasterosteus aculeatus): Influence of body size and temperature
Source: PLoS One. 2018 Apr 3;13(4):e0194932. doi: 10.1371/journal.pone.0194932 (PMC5882091; doi:10.1371/journal.pone.0194932)
Supplement: S2 Table — The model was constructed, considering size as continuous covariate, and sex as factor. (DOCX) [file pone.0194932.s002.docx]

# Supporting information

**S2 Table. ANCOVA results of gut morphometric parameters in sticklebacks exposed at 0, 60, 120, 180 and 240 days to a temperature-photoperiod cycle. The model was constructed, considering size as continuous covariate, and sex as factor.**

| Time condition | Covariate and factor | Relative gut length (RGL) | | | | Relative gut mass (RGM) | | | | Zihler’s index (ZI) | | | |
| --- | --- | --- | --- | --- | --- | --- | --- | --- | --- | --- | --- | --- | --- |
|  |  | d.f. | SS | *F* | *p* | d.f. | SS | *F* | *p* | d.f. | SS | *F* | *p* |
| Day 0 | Size | 1 | 0.0001 | 0.014 | 0.907 | 1 | 0.0000 | 1.421 | 0.239 | 1 | 544.7 | 180.6 | **<0.0001^***^** |
|  | Sex | 1 | 0.0004 | 0.038 | 0.846 | 1 | 0.0000 | 0.896 | 0.348 | 1 | 0.300 | 0.107 | 0.745 |
|  | Size:Sex | 1 | 0.0008 | 0.082 | 0.776 | 1 | 0.0000 | 0.944 | 0.336 | 1 | 0.000 | 0.000 | 0.984 |
| Day 60 | Size | 1 | 0.0166 | 2.405 | 0.127 | 1 | 0.0012 | 28.33 | **<0.0001^***^** | 1 | 490.4 | 179.622 | **<0.0001^***^** |
|  | Sex | 1 | 0.0021 | 0.299 | 0.587 | 1 | 0.0000 | 0.299 | 0.586 | 1 | 7.700 | 2.804 | 0.100 |
|  | Size:Sex | 1 | 0.0007 | 0.104 | 0.749 | 1 | 0.0000 | 1.301 | 0.259 | 1 | 0.200 | 0.079 | 0.780 |
| Day 120 | Size | 1 | 0.0347 | 3.981 | 0.051 | 1 | 0.0000 | 1.147 | 0.289 | 1 | 101.6 | 43.048 | **<0.0001^***^** |
|  | Sex | 1 | 0.0136 | 1.554 | 0.218 | 1 | 0.0000 | 0.019 | 0.890 | 1 | 1.850 | 0.784 | 0.380 |
|  | Size:Sex | 1 | 0.0212 | 2.425 | 0.125 | 1 | 0.0000 | 0.099 | 0.755 | 1 | 0.010 | 0.004 | 0.952 |
| Day 180 | Size | 1 | 0.0078 | 1.576 | 0.217 | 1 | 0.0000 | 0.005 | 0.944 | 1 | 68.31 | 68.775 | **<0.0001^***^** |
|  | Sex | 1 | 0.0038 | 0.763 | 0.388 | 1 | 0.0000 | 1.071 | 0.306 | 1 | 0.500 | 0.503 | 0.482 |
|  | Size:Sex | 1 | 0.0055 | 1.113 | 0.298 | 1 | 0.0002 | 3.320 | 0.075 | 1 | 0.190 | 0.195 | 0.662 |
| Day 240 | Size | 1 | 0.0030 | 0.853 | 0.366 | 1 | 0.0889 | 3.888 | 0.061 | 1 | 14.30 | 26.48 | **<0.0001^***^** |
|  | Sex | 1 | 0.0004 | 0.133 | 0.719 | 1 | 0.0038 | 0.168 | 0.685 | 1 | 0.014 | 0.026 | 0.873 |
|  | Size:Sex | 1 | 0.0021 | 0.593 | 0.450 | 1 | 0.0216 | 0.943 | 0.342 | 1 | 5.438 | 10.06 | **0.004^**^** |

d.f.: Degrees of Freedom; SS: Sums of Squares; *F*: ANCOVA F test; *p*: *p*-value.
