# Supplementary figures and images for: Digestive enzymes and gut morphometric parameters of threespine stickleback (Gasterosteus aculeatus): Influence of body size and temperature
Source: PLoS One. 2018 Apr 3;13(4):e0194932. doi: 10.1371/journal.pone.0194932 (PMC5882091; doi:10.1371/journal.pone.0194932)

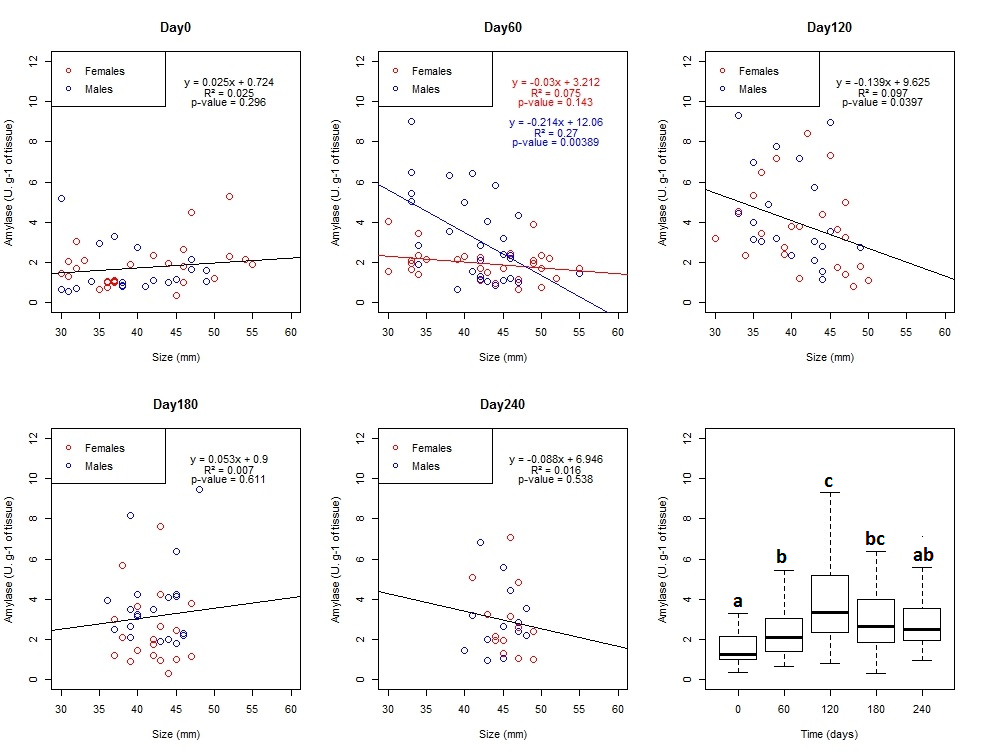

Supplement: S1 Fig — The model was constructed, considering size as continuous covariate, and sex as factor. Note that a single regression line was plotted in absence of sex effect. Males were plotted in blue, and females in red. (TIF) [file pone.0194932.s003.tif]

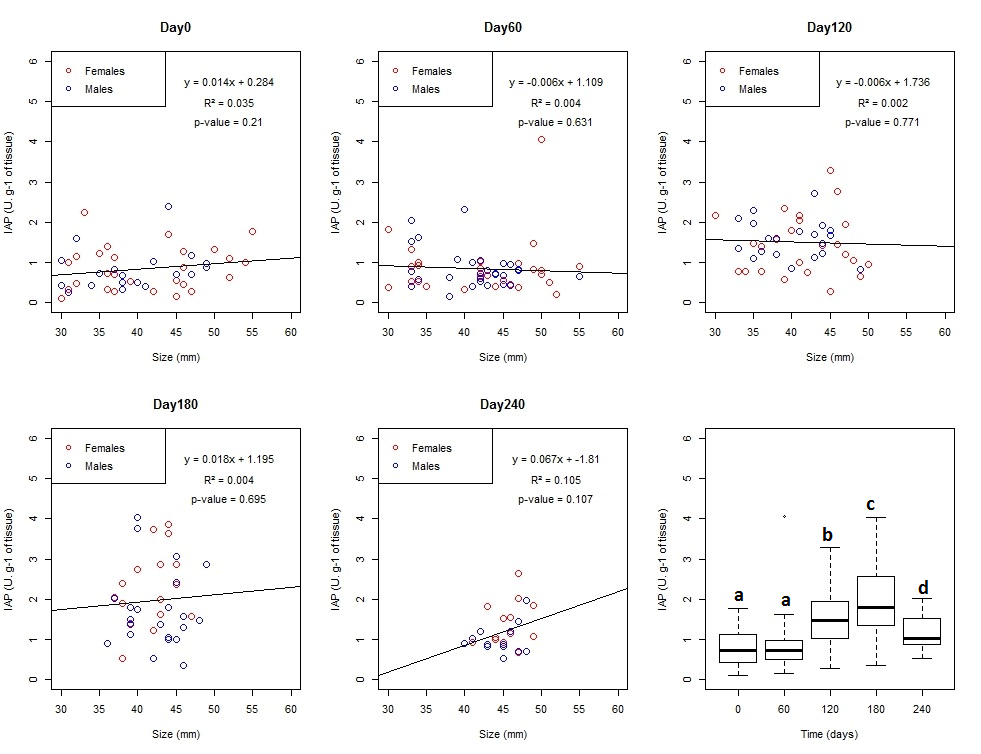

Supplement: S2 Fig — The model was constructed, considering size as continuous covariate, and sex as factor. Note that a single regression line was plotted in absence of sex effect. Males were plotted in blue, and females in red. (TIF) [file pone.0194932.s004.tif]

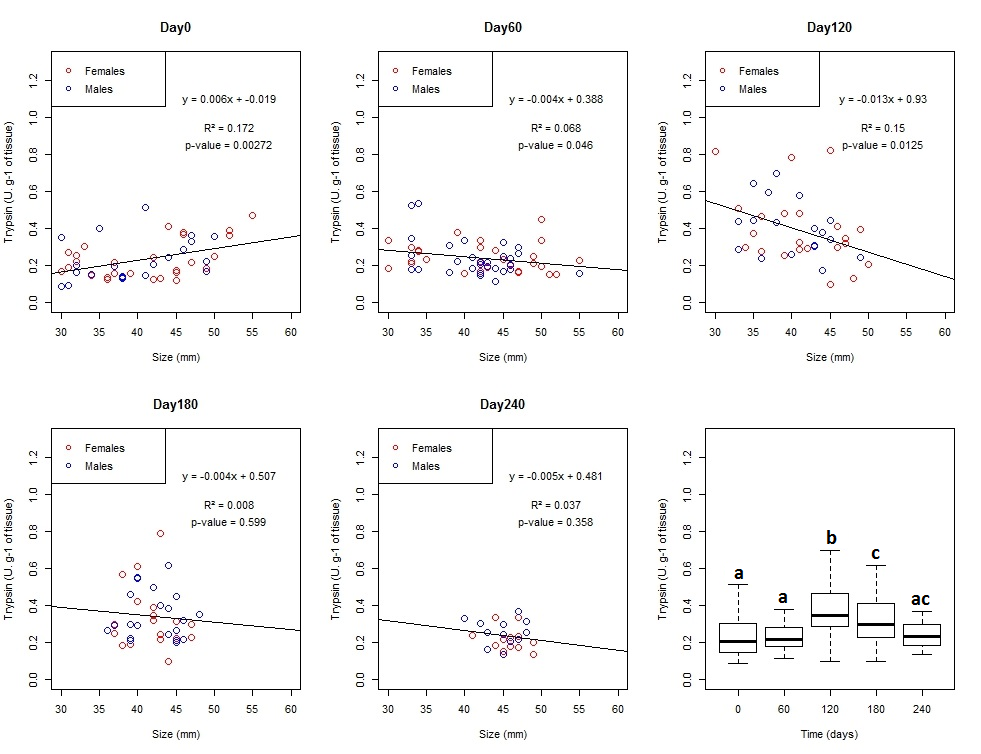

Supplement: S3 Fig — The model was constructed, considering size as continuous covariate, and sex as factor. Note that a single regression line was plotted in absence of sex effect. Males were plotted in blue, and females in red. (TIF) [file pone.0194932.s005.tif]
